# Factors causing work related stress and strategies for stress management: a study of working women in private and public sectors in the Indian context

**DOI:** 10.3389/fgwh.2025.1597409

**Published:** 2025-05-16

**Authors:** Saranya Chandrasekaran, Rajakumar Guduru, Saranraj Loganathan

**Affiliations:** ^1^ Department of English, Mepco Schlenk Engineering College, Sivakasi, Tamil Nadu, India; ^2^ School of Humanities, Social Sciences & Management, Indian Institute of Technology, Bhubaneswar, Odisha, India; ^3^ Department of English, Mepco Schlenk Engineering College, Sivakasi, Tamil Nadu, India

**Keywords:** work-related stress, stress management, working women, private and public, India

## Abstract

In the present context, various underlying social factors such as workload, lack of support, job insecurity, work-life imbalance, mental health, gender bias, financial pressure, harassment, and discrimination cause stress to women. Therefore, the main purpose of this study is to identify some of the pertinent factors affecting working women's mental health and physical health in the work place. The study also aims at finding strategies for coping with stress. A cross-sectional research design and simple random sampling method were used in this study. This study was conducted between the women employees of both public and private sector institutes employed in the fields of teaching, banking, nursing, clerical and IT professionals across the state of Tamil Nadu, India. The study adopted the survey method and the data were collected by administering questionnaires to 200 (100 public sector and 100 private sector) participants through online mode and were analysed both qualitatively and quantitatively. The results of the study showed that the majority of the women (32.5%) experienced stress when evaluated performance by superiors and given negative feedback, by their employers. The analysis also showed that the most effective strategy for stress management was spending time with children, with an average rank of 3.55 whereas doing yoga and meditation has the lowest average rank of 2.65, indicating that respondents rarely use this strategy for managing stress. The study offers some measures to support their mental and physical health and to ensure equal opportunities for all. Finally, AI-driven solutions are recommended to foster a dynamic and responsive environment that empowers working women to proactively manage stress, boost mental well-being, and improve overall productivity.

## Introduction

The world is going through many drastic shifts which cause stress to humans around the world. Stress affects people at all age levels and at all aspects of day-to-day life. It seems stress is natural response to all humans in a way it helps in coping with certain challenging situations in life. One of the most cited definitions on stress is by the World Health Organization (WHO) which states that stress is “a state of worry or mental tension caused by a difficult situation”. Similarly, UNICEF states as “a common feeling we get when we feel under pressure, overwhelmed or unable to cope.” To some extent, everyone is stressed. However, how we respond to stress has a significant impact on our general well-being. The definitions of stress in general, mostly stress is perceived as personal as people experience stress in different ways. Stress can impact people positively by pushing them to achieve their goals. However, too much stress can impact people negatively affecting health, mood, and mental well-being. Therefore, it is important to know and understand types of stress. Weariness, nausea, vomiting, fainting, high blood pressure, tightness in the muscles or jaw clenching, gastrointestinal or digestive difficulties, and a weak immune system are examples of physical signs of stress. Stress can also lead to severe heal issues like depression and panic attacks. Job related stress is growing worldwide which affects peoples’ health and also the effectiveness and productivity of the organization which they work for. Job related stress is caused by factors such as workload, conflict with colleagues, job insecurity, and stagnant, frequent job change. Job-related stress manifests itself in behavioural symptoms such as an increase in sick days or inactivity, aggression, reduced creativity and initiative, a drop in work performance, challenges with interpersonal relationships, mood swings and irritability, less tolerance of frustration and impatience, boredom, and isolation. Nevertheless, it is observed that most women hesitate to disclose the factors causing stress.

The present study aims at identifying factors causing stress in working women as well as finding the common stress management strategies which these women adopt in day-to-day life. Presently, most companies, public and private sector, have been evolving to become more flexible and employee friendly in terms of providing facilities. However, the common observation is that women employees are still facing stress at various levels in various work-related contexts. Thus, this study is important for few reasons that it helps women to manage stress in a healthier way by helping them become aware of different factors which cause stress in public and private sector work places. This study also will help women understand various stress management activities and strategies in work place. Most importantly, the study also creates a sense of awareness and responsibility among working women to lead a stress-free life.

### Objectives of the study

The aim of this study is to identify some of the most common factors causing stress for women in the work environment. The objectives of the study is

• To identify the level of stress faced by working women and the sources factors of stress in working women.
• To understand different strategies adopted by working women to reduce their work-related stress.

### Hypothesis

Null Hypothesis H_0_1: There is no significant difference between problem description stresses and Strategies adopted for managing stress.

### Literature review

Despite the increased research on the factors causing stress in women in the workplace, there still remain specific stressful situation in daily life such as less salary, gender discrimination, time pressure and mental health. Based on the occurrences of stressors few studies have been conducted in the Indian context. Hence, we have done a review of some of the past research studies which focused on the factors causing stress in the working women. Influencers of Stress Level among Working Women in Chennai City, found that feeling of inequality and lack of time management and planning are major factors causing the personal stress among working women (1). The working women feel more stress than working men due to work and home stress, handling children and office work (2). According to Thirumaleswari (3) factors like ill treatment, gossip, and criticism at work places are the major causes for burnout, and they lead to personal, group, family and social impact and interventions. Working women in both private sector and public sector were found to be more stressful due to their dual role whereas women working in private sector were found to be higher stressed than women in government sector (4). However (5), Jindal found that mostly working women were unable to balance their work and personal life irrespective of the sector they are into, the age group they belong to, the number of children they have, income and their occupation. Out of 98(100%) working women only 44(44.9%) women have balanced in their work and life. In a similar study, stress is very common in people and is hard to avoid. Stress is more experienced in day-to-day living by working women than non-working women (6). In addition, employees’ mental health becomes an important path to explain the relationship mechanism between work stress and employee performance, which is revealed in this study using a stress–psychological state–performance framework (7) (8). Abdou found that high levels of work-related stressors can spill over into employees’ family lives, creating a conflict between work and family responsibilities. Further, work stress directly influences psychological distress, part of its impact is directed through its effect on work-family conflict. The major finding of the research showed that role overload has a significant impact on job stress as the women have to play various roles in a family as well as in the organization (9). Moreover, they also found that it was for women to perform all roles with similar commitment. The study stated that role overload led to fatigue, stress, and dissatisfaction and is negatively associated with job satisfaction. In highlighting the positive relationship between stress in women and family difficulties, increase in work-family conflict leads to increase in work stress and vice versa in married women. The study also concluded that women professionals with high job demands were more prone to experience work-family conflict and work-related stress (10). Similarly, the major factors influencing the work life balance of women professionals in India such as role conflict, lack of recognition, organizational politics, gender discrimination, elderly and children care, quality of health, problems in time management and lack of proper social support (11). It was identified that in the hospitality sector, three significant stressors—role conflict, ambiguity, and overload—contribute to work-related stress among employees. The previous research studies have explored women's stress under specific situations (12).

A Study on Organisational Role of Stress among Women Working in Private Colleges in Mangalore using ORS Scale, women are entering into corporate world to earn their living, so they recommend that organisations need to take care of basic stress causing agents which might lead to increase in rate of attrition among women working in the organisations (13). Likewise, It was observed that occupational stress can be managed through practicing meditation, physical work, morning walk and many more things to remove theses stresses (14). Mohammad Amiri, investigated occupational stress among IT employees using the Occupational Stress Index Scale. The research identifies primary stressors such as heavy workloads, tight deadlines, job insecurity, inadequate compensation, and limited career advancement opportunities. These factors contribute to both physical and psychological stress, manifesting as fatigue, anxiety, and decreased job satisfaction (15). Dharmaraja and Basavaraj explored the work-life balance challenges faced by women employees in India. The study highlighted issues such as role overload, time management problems, and lack of social support, leading to stress (16). From the light of literature review, it has been identified that no qualitative studies have been conducted on work related stress and strategies for stress management among working women in both private and public sectors.

## Methodology

A cross-sectional research design and simple random sampling method were used in this study. A qualitative study was conducted using a questionnaire, which was collected through online.

### Participants

The sample size is 200 women employees (100 from the public sector and 100 from the private sector), who are working in the fields of teaching, banking, nursing, clerical and IT professionals.

### Tools

The research tools such as simple percentage, Karl pearson correlation coefficient test, *T*-test for two independent mean, one way classification ANOVA and weighted average method were employed to analyse the data with reference to the selected objectives of the study.

### Data collection

The primary method of data collection was adopted in this study. Data were collected through online questionnaire under three segments: (1) Personal Details, (2) Problem Description, (3) Adopted strategies to manage stress level. The questionnaire was administered to 200 women employees belonging to both public and private sectors in various places in Tamil Nadu, India.

The collection of opinions of women employees of public and private sectors constituted the survey method in the study.

The sample was also designed to be geographically diverse, including participants from various districts and urban/rural regions across Tamil Nadu, India. Although there was no explicit stratification by location, the online mode of data collection facilitated outreach to a wide demographic base, thereby minimizing geographic bias. Similarly, while age was not a stratification criterion, the random sampling approach helped ensure a natural variation in age distribution across the sample. Women who were unemployed, working outside the listed fields, living outside Tamil Nadu, or who submitted incomplete or duplicate responses were not included in the study. To reduce selection bias, participants were chosen through random sampling. The online survey was shared through professional groups, emails, and social media to reach a wide range of people. No rewards were given for taking part, which helped make sure people joined the study for genuine reasons. Consent was taken, and all responses were kept private to make participants feel comfortable and safe sharing their views. The Internal reliability Cronbach's alpha is 0.910 for 23 items, which is excellent.

### Data analysis and interpretation

Table 1 shows the distribution of the population and two samples (Sample I and Sample II) across four categories: Age Group, Marital Status, Employment Status, and Family Status. From the above-mentioned table, it is identified that the highest number of respondents who took part in this study are youngsters those who belongs to 25–30 age category. Obviously, the married women are the highest respondents. In terms of employment status, the majority of respondents have less than 5 years of experience, particularly in Sample II. Moreover, most of the private sector women employees participated in the study belong to nuclear family with the highest percentage of Sample II.

**Table 1:** Women employee's personal details.

Category		Population (*N*)	Sample I (n_1_)	Sample II (n_2_)
Age group	25–30	41.5%	29%	54%
	31–35	29%	27%	31%
	36–50	20%	26%	14%
	50 above	9.5%	18%	1%
Marital status	Spinster	31%	28%	34%
	Married	64.5%	65%	64%
	Divorced or widowed	4.5%	7%	2%
Employment status	Below 5 years	49%	40%	58%
	5–10 years	26.5%	26%	27%
	Above 10 years	24.5%	34%	15%
Family status	Joint family	45%	49%	41%
	Nuclear family	55%	51%	59%

Source: author's own work. Population *N* = 200 (Women employee in Public Sector + Women employee in Private Sector). Sample I n_1_ = 100 (Women employee in Public Sector). Sample II n_2_ = 100 (Women employee in Private Sector).

The Table 2 illustrates the results of the survey on the causes of workplace stress among working women. The highest respondents were reported they have been undergoing stress in moderate level among the overall population, Sample—I and Sample—II as well in the categories of promotions and additional responsibilities, working overtime or long hours, unavailability of leave taking for emergency purposes, pay disparity, job insecurity threads, inadequate facilities in working environment/circumstances and lack of opportunity for growth and advancement. Women those who are working in public sector undergoes the highest level of stress in terms of evaluating performance by superiors and their negative feedback and work pressure from higher authorities as reported in this study.

**Table 2:** Factors of workplace stress among working women.

Factors	Category	Always	Sometimes	Never
Promotions and additional responsibilities	Population (*N*)	20.5%	44.5%	35%
	Sample I (n_1)_	16%	47%	37%
	Sample II (n_2)_	25%	42%	33%
Working overtime or long working hours	Population (*N*)	19.5%	55.5%	30%
	Sample I (n_1)_	16%	57%	27%
	Sample II (n_2)_	23%	54%	33%
Evaluating performance by Superiors and their negative feedback	Population (*N*)	32.5%	37%	30.5%
	Sample I (n_1)_	42%	38%	20%
	Sample II (n_2)_	23%	36%	41%
Work pressure from higher authorities	Population (*N*)	21%	40.5%	38.5%
	Sample I (n_1)_	20%	34%	46%
	Sample II (n_2)_	22%	47%	31%
Unavailability of leave taking for emergency purposes	Population (*N*)	20%	52%	28%
	Sample I (n_1)_	17%	55%	28%
	Sample II (n_2)_	23%	49%	28%
Pay disparity	Population (*N*)	19%	49.5%	31.5%
	Sample I (n_1)_	15%	50%	35%
	Sample II (n_2)_	23%	49%	28%
Job insecurity threads	Population (*N*)	17.5%	43%	39.5%
	Sample I (n_1)_	13%	39%	48%
	Sample II (n_2)_	22%	47%	31%
Inadequate facilities in working environment/ circumstances	Population (*N*)	25.5%	38.5%	36%
	Sample I (n_1)_	23%	39%	38%
	Sample II (n_2)_	28%	38%	34%
Lack of opportunity for growth and advancement	Population (*N*)	24%	39%	37%
	Sample I (n_1)_	23%	35%	42%
	Sample II (n_2)_	25%	43%	32%

Source: author's own work.

Table 3 presents coping strategies adopted by women employees to manage workplace stress. The most priorities are given for Visiting temples and prayers, arranging family trips in working Environment, Spending time with children respectively by the respondents of all categories in order to manage workplace stress.

**Table 3:** Stress coping strategies adopted by women employees.

Strategies	Category	Very often	Often	Sometimes	Rarely	Never
Visiting temples and prayers	Population (*N*)	30.5%	23.5%	26.5%	8%	11.5%
	Sample I (n_1)_	28%	27%	32%	4%	9%
	Sample II (n_2)_	33%	20%	21%	12%	14%
Arranging family trips in working Environment	Population (*N*)	30.5%	25%	19%	21.5%	16%
	Sample I (n_1)_	17%	28%	23%	19%	13%
	Sample II (n_2)_	20%	22%	15%	24%	19%
Meeting friends or relatives	Population (*N*)	22.5%	17.5%	28%	20%	12%
	Sample I (n_1)_	18%	10%	29%	27%	16%
	Sample II (n_2)_	27%	25%	27%	13%	8%
Doing yoga and meditation	Population (*N*)	16%	15.5%	19%	16.5%	33%
	Sample I (n_1)_	15%	11%	20%	17%	37%
	Sample II (n_2)_	17%	20%	18%	16%	29%
Making annual holidays (compulsory)	Population (*N*)	27.5%	18.5%	19.5%	13%	21.5%
	Sample I (n_1)_	27%	17%	22%	12%	22%
	Sample II (n_2)_	28%	20%	17%	14%	21%
Doing recreational activities	Population (*N*)	16.5%	20%	27%	18%	18.5%
	Sample I (n_1)_	16%	21%	27%	20%	16%
	Sample II (n_2)_	17%	19%	27%	16%	21%
Watching television	Population (*N*)	19.5%	23%	29.5%	15.5%	14%
	Sample I (n_1)_	23%	23%	31%	13%	10%
	Sample II (n_2)_	16%	23%	25%	18%	18%
Shopping	Population (*N*)	25%	21%	24%	21%	9%
	Sample I (n_1)_	25%	27%	27%	14%	7%
	Sample II (n_2)_	25%	15%	21%	28%	11%
Spending time with children	Population (*N*)	32%	22.5%	24%	11.5%	10%
	Sample I (n_1)_	32%	20%	28%	9%	11%
	Sample II (n_2)_	32%	25%	20%	14%	9%

Source: author's own work.

**Table 4:** Karl Pearson correlation coefficient test.

Variables	Population (public & private)	Sample I public sector	Sample II private sector
Correlation value	r = 0.23	r = 0.33	r = 0.14
Conclusion	+ ive correlation	+ ive correlation	+ ive correlation
Relationship between problem description & strategies adopted for managing stress	Weak	Weak	Very weak

Source: author's own work.

## Findings

### Karl Pearson correlation coefficient test

The Karl Pearson Correlation Coefficient measures the strength and direction of a linear relationship between two variables. In this case, the variables are the causes of stress and the strategies for managing stress. The correlation values for three samples (population, sample I, and sample II) are provided in Table 4.

A positive correlation means that as one variable increases, the other tends to increase as well.

However, the correlation values suggest a weak to very weak relationship. The highest value (r = 0.33) indicates a weak correlation, while the lowest (r = 0.14) shows a very weak correlation. This means there is some positive link between the two variables, but the relationship is not strong. On the whole, the results suggest a positive but weak relationship between the causes of stress and the strategies for managing stress.

### *t*-test for two independent mean

From the Table 5, it is observed that the H_1_ is accepted for both Sample I and Sample II, and there is evidence to suggest that the mean population for women in public sector is different from the mean population for women in private sector.

**Table 5:** *t*-test for two independent mean.

Variables	Population public & private sectors	Sample I public sector	Sample II private sector
t value	−4.76469	−4.41027	−2.37962
*P* value	Less than 0.00001	0.00017	0.18281
Conclusion	Ha1 is accepted	Ha1 is accepted	Ha1 is accepted
Reason	*P* < 0.05	*P* < 0.05	*P* < 0.05

Source: author's own work.

### One-way classification (ANOVA table)

Table 6 presents the results of an ANOVA test comparing three groups: Since all *P*-values are below 0.05, we reject the H_0_1 and accept the H_a_1, concluding that there are significant differences between the groups.

**Table 6:** One-way classification (ANOVA table).

Variables	Population public & private sectors	Sample I public sector	Sample II private sector
F value	22.70	19.45	5.66
*P* value	Less than 0.00001	0.00017	0.018281
Conclusion	Ha1 is accepted	Ha1 is accepted	Ha1 is accepted
Reason	*P* < 0.05	*P* < 0.05	*P* < 0.05

Source: author's own work.

The Weighted Average Method is used to calculate the weighted average score for each factor in the Table 7. Based on the analysis, we can see that the factor with the highest weighted average Problem Description is “Evaluating performance by Superior and their negative feedback,” with a rank of 1. This indicates that this factor has the greatest impact on job satisfaction, according to the survey respondents. And the factor “Work pressure from higher authorities” has the least impact with weighted rank of 8. This suggests that employees may not consider this pressure as a major contributor to their overall stress levels in the workplace compared to other factors.

**Table 7:** Weighted average method: problem descriptions.

Problem descriptions	Always	Sometimes	Never	Average	Rank
Promotions and additional responsibilities	41	89	70	2.71	Rank 7
Working overtime or long working hours	39	101	60	2.79	Rank 3.5
Evaluating performance by superior and their negative feedback	65	74	61	3.04	Rank 1
Work pressure from higher authorities	42	81	77	2.65	Rank 8
Unavailability of leave taking for emergency purposes	40	104	56	2.84	Rank 2
Pay disparity	38	99	63	2.75	Rank 5
Job insecurity threads	35	86	79	2.56	Rank 9
Inadequate facilities in working environment/circumstances	51	77	72	2.79	Rank 3.5
Lack of opportunity for growth and advancement	48	78	74	2.74	Rank 6

Source: author's own work.

The Table 8 provides data on the frequency of adoption of different strategies for managing stress among the respondents. According to the study, the most effective strategy for managing stress is spending time with children, indicating that respondents frequently use this strategy to manage stress. Doing yoga and meditation has the lowest average rank, indicating that respondents rarely use this strategy for managing stress. Overall, the data suggest that spending time with children, visiting temples and prayers, shopping, making annual holidays compulsory, and meeting friends or relatives are the most effective strategies for managing stress, while doing yoga and meditation is the least effective.

**Table 8:** Weighted average method: strategy adopted for managing stress.

Strategy adopted for managing stress	Very often	Often	Some times	Rarely	Never	Average	Rank
Visiting temples and prayers	61	47	53	16	23	3.54	Rank 2
Arranging family trips in working environment	40	47	38	43	32	3.1	Rank 7
Meeting friends or relatives	45	35	56	40	24	3.19	Rank 4
Doing yoga and meditation	32	31	38	33	66	2.65	Rank 9
Making annual holidays (compulsory)	55	37	39	26	43	3.18	Rank 5
Doing recreational activities	33	40	54	36	37	2.98	Rank 8
Watching television	39	43	59	31	28	3.17	Rank 6
shopping	50	42	48	42	18	3.32	Rank 3
Spending time with children	64	45	48	23	20	3.55	Rank 1

Source: author's own work.

## AI–driven tools for effective stress reduction

AI technologies and machine learning (ML) algorithms provide personalized, real-time stress management solutions for working women by integrating data from wearable devices, stress-monitoring applications, and workplace management platforms. These AI systems analyse critical physiological indicators such as heart rate variability, sleep patterns, and physical activity levels to detect early signs of stress, allowing for the recommendation of tailored interventions, including guided meditation, breathing exercises, or brief restorative breaks. By utilizing natural language processing (NLP), AI-powered chatbots facilitate therapeutic conversations, offering emotional support and assisting users in developing effective coping mechanisms. Chatbots such as Wysa and Woebot offer emotional support and therapeutic conversations, creating a safe space for managing mental health. These chatbots also conduct sentiment analysis on workplace communications to identify stress-inducing factors like negative feedback, workplace harassment, and gender bias. Time management tools like Google Calendar and Asana help prioritize tasks and automate reminders, while AI-powered wellness apps like Calm and Headspace provide guided meditation and stress-relief techniques tailored to individual needs. Working women can use AI-driven fitness trackers like Fitbit monitor physical activity, sleep, and stress levels, offering actionable insights to maintain well-being. In addition, AI employs predictive analytics to foresee potential workload imbalances and suggest optimal task distribution, prioritization, and time management, seamlessly integrating with task management tools like Asana or Trello. Through adaptive learning models, the system evolves by learning from user interactions, behavioural patterns, and continuous feedback, fine-tuning its recommendations to better suit individual needs. These AI-driven solutions foster a dynamic and responsive environment that empowers working women to proactively manage stress, boost mental well-being, and improve overall productivity, thus creating a more supportive, balanced, and efficient workplace.

## Discussion

Effective stress management is vital for working women to maintain a healthy work-life balance while addressing workplace challenges. Especially for working women, self-care is fundamental, including adequate sleep, a balanced diet, regular exercise, and relaxation practices like mindfulness. Further, time management helps them to reduce overwhelm through realistic goal-setting, task prioritization, and setting boundaries by delegating responsibilities and limiting over commitment. Women who are working in private sector experienced more emotional stress than physical stress (1). Therefore, they are involving themselves in rejuvenation activities such as spending time in nature, pursuing hobbies, or journaling provide emotional refreshment and relaxation techniques like aromatherapy and progressive muscle relaxation offer immediate relief from stress. And also, building resilience through reframing challenges, practicing gratitude, and engaging in joyful activities such as comedies or volunteering fosters a positive outlook and emotional strength allow women to manage stress effectively while enhancing their overall well-being. Based on the employee industry, working women stress varied (3).

Organizational stress leads to poor productivity (13) thus organisation can further support their female workforce by implementing wellness programs, flexible policies, and stress management workshops, creating an inclusive environment that promotes productivity and personal growth. By creating a supportive and inclusive learning environment, companies can empower women to prioritize their mental and physical health, equipping them with tools to thrive both at work and in their personal lives. This initiative not only benefits the individuals but also contributes to a more productive and harmonious workplace culture. Further, the study highlights the significance of personal and family-oriented strategies in managing work-related stress among women. Especially, married women maintained better work-life balance than the unmarried women (7, 16). They are visiting temples and engaging in prayers serve as effective spiritual coping mechanisms, offering emotional solace and mental clarity amidst workplace pressures. Employees who are working in corporate industries had too much of stress thus it lead to health issues and disturb their family members (14), so they are organizing family trips provides a much-needed break from the routine, fostering relaxation and rejuvenation while strengthening familial bonds. In addition, spending quality time with children further enhances emotional well-being, serving as a source of joy and purpose that alleviates stress. Among married women, the increase in work conflict leads to stress and increase in family conflict (10).

## Conclusion

Women seem to experience more stress as they involve themselves in all walks of life. Hence, it is important that women are aware of stress management strategies so that they can avoid situations like burnout, negativity or unproductivity in the work places. Moreover, research shows that when women are given the opportunity to work in a less stressful setting, their motivation increases and they feel more enthusiastic about their jobs. Stress can be very much managed. However, besides self-care, it is the responsibility of the organization to ensure a positive, thriving, and meaningful engagement. Some of the useful stress management strategies that could be implemented in the workplace are like: adopting to relaxation techniques to enhance the vitality and quality of life; building up stronger and healthier stress-free culture; creating a sense of feel good about quality of life and well-being; sensitizing how to balance work-life and personal life, and so on. Most importantly, all working women should take tangible steps to prioritize stress reduction. By creating a supportive and inclusive learning environment, companies can empower women to prioritize their mental and physical health, equipping them with tools to thrive both at work and in their personal lives.

### Limitations of the study

The present study is limited to employees of working women the particular department of public and private sectors. Further, this survey is taken only from women employees belong to Tamil Nadu, India.

## Data Availability

The original contributions presented in the study are included in the article/Supplementary Material, further inquiries can be directed to the corresponding author.
